# Aneurysm

**Published:** 1908-05-09

**Authors:** 


### ANEURYSM.

### FANTASTIC SURGICAL TREATMENT.

It has already been stated that the only proper and justifiable treatment of aneurysm is some form of ligature of the vessel from which it springs; and a short account has been given of the methods which have been devised to this end. It has also been explained that ligature is impracticable in a large number of cases for anatomical reasons on account of the situation of the aneurysm. But there are also other contra-indications to this method of treatment; thus it is inadvisable to proceed to ligature in a patient of an advanced age or in one whose general arterial system is extensively damaged by disease. The co-existence of morbus cordis or of Bright's disease should also deter one from active surgical measures. And again, if there is more than one aneurysm a single operation will not cure the patient. Nor should an operation be undertaken if the pressure of an aneurysm has already eroded bones or invaded joints. And, finally, it is hopeless to expect that ligature of the vessel will cure an aneurysm unless it can be previously shown that occlusion of the main vessel by digital pressure can arrest pulsation in the sac.

In view of these facts an almost countless number of alternative procedures has been devised. The oldest of these, and the most interesting, if only from the historical point of view, is treatment by compression. In the days before the introduction of antiseptic methods when any operation on the trunk of an artery was likely to be followed by dire results this was often practised in preference to open operation. Digital compression was most commonly used because it was convenient and easily applied; but it had the disadvantage that it was only applicable in certain situations where the main trunk lay superficially, and had behind it some resisting structure against which it could be compressed. The artery was pressed with the thumb or finger until there was no pulsation in the aneurysm, but the method was found to be excessively tiring, and mechanical assistance was obtained by the superposition of a bag of shot or a sandbag. Even then it was found that relays of assistants were necessary, and clinical clerks or dressers were told off for this duty. Almost every surgeon of a certain seniority can recall such a procedure as among his most vivid and interesting reminiscences, but to the present generation the method has only a historical interest.

Many forms of instrumental compression were therefore devised with a view to overcoming this difficulty; but they all suffer from the disadvantage of a mechanical contrivance compared to the human being, that it has no brain, and therefore, however exactly and perfectly applied, it acts without discrimination. It is unnecessary as well as unprofitable to describe all the varieties of compressor which have been evolved by the ingenuity of successive generations of surgeons. Few of them are now seen outside museums, and only two deserve even passing mention. One is Lister's aorta compressor, and the other is Davy's rectal lever. Both were devised for the treatment of aneurysms of the lower extremity. Lister's compressor consists of a flat pad which is applied to the back, connected to a bar which goes round the body, through the anterior extremity of which a second pad is screwed down on to the anterior abdominal wall with just sufficient force to occlude the abdominal aorta. The application of this instrument is not only excessively painful, but is recorded to have done serious damage, and on one occasion even to have ruptured the pancreas. Davy's lever is a solid rod, which is inserted into the rectum and manipulated so that its end compresses the common iliac artery against the pelvic brim.

Another method of producing compression which is even more painful than the last is that of bringing about such acute flexion of the limb—*e.g.* at the knee-joint—that the pulse in the artery is obliterated below that point. Since the Listerian era began, such methods are no longer considered worthy of the name of surgery.

Compression is therefore an admittedly unsatisfactory manœuvre, whatever means are adopted to occlude the artery, and a series of methods not less ingenious has been invented with the object of producing coagulation in the sac. The principle is the same in all, though there are differences in the means employed.

MacEwen of Glasgow has recorded cases in which coagulation was induced by inserting a needle into the sac just so far that the point was in contact with the endothelium on the far side. The movements of the blood-stream cause oscillation of the needle, so that the endothelium is scratched, and the slight injury so produced is the starting-point of a clot. Others have produced coagulation by electrical methods. A needle, which is the positive pole, is inserted into the aneurysm, while the negative pole is applied to the skin. Clotting is said to take place round the positive pole.

Much ingenuity has also been expended upon inventing instruments by which foreign bodies can be inserted into the aneurysm. Horse-hair has been employed in this way. The results of this treatment are not uniformly good, but it would seem that the least harmful method is to introduce a small quantity of fine gold wire through a Southey's canula. The principle, of course, is mechanical, the gold wire forming a basis on which a clot may form.

Good results were at one time reported from the subcutaneous injection of a dilute solution of gelatine, but the pioneers of the method were discouraged by the difficulty of obtaining a sterile solution, and several cases occurred of infection with the bacillus tetani, which were directly attributable to the gelatine solution. It has since been shown that the value of the method probably depended on the fact that the gelatine contained calcium salts, and that if these were previously removed the gelatine itself had no effect upon the aneurysm at all. This is borne out by the writer's experience. A small series of cases of aneurysm was treated by decalcified gelatine. No harmful effects followed, but it could not honestly be said that any improvement did either. After a time calcium chloride was simultaneously given by the mouth, and it certainly seemed to set up coagulation in some cases.

